# The Accumulation of Chemical Components of Volatile Oil in *Cinnamomum migao* Fruit Is Related to Endophytic Fungi

**DOI:** 10.1002/ece3.71548

**Published:** 2025-06-09

**Authors:** Shuang‐Yan Hu, Lan Zhang, Xin Yang, Qing‐Wen Sun, Jing‐Zhong Chen

**Affiliations:** ^1^ College of Pharmacy Guizhou University of Traditional Chinese Medicine Guiyang People's Republic of China; ^2^ Guizhou Traditional Chinese Medicine National Medicinal Materials Germplasm Resources Preservation and Evaluation Engineering Research Center Guiyang People's Republic of China; ^3^ Guizhou Key Laboratory for raw Material of Traditional Chinese Medicine Guiyang Guiyang People's Republic of China; ^4^ Guizhou Academy of Forestry Sciences Guiyang People's Republic of China

**Keywords:** differential components, Lauraceae, metabolites of endophytic fungi, microbial diversity

## Abstract

Plants naturally host a variety of microbial communities, which significantly affect their health and productivity. *Cinnamomum migao* is a rare and endangered medicinal plant endemic to Southwest China. However, the related research on endophytic fungi of *C. migao* has been neglected, especially in the fruit, and the interaction between microorganisms in *C. migao* fruit and its effect on the productivity of *C. migao* host plants is still unclear. In this study, the endophytic fungi of *C. migao* fruits in Guizhou, Yunnan, and Guangxi of Southwest China were isolated, purified and cultured, sequenced, and the metabolites of endophytic fungi and the contents of volatile oil components in *C. migao* fruits were determined and analyzed. The results showed that 476 strains of endophytic fungi (3 class, 6 order, 13 families, 20 genera) were isolated and identified from *C. migao* fruits, which showed that there were some differences in the composition of endophytic fungi in *C. migao* fruits from different producing areas. Five differential components—γ‐eucalyptol, bulnesol, bornyl acetate, spathulenol, and linalool were—selected from the essential oils of *C. migao* fruits, showing both positive and negative correlations with the different locations; these metabolites both promoted and inhibited each other. Additionally, eight cultivable endophytic fungal strains from *C. migao* fruits were found to share the same chemical composition in their metabolites as the essential oils, and there were also different positive and negative correlations with the origins.

## Graphical abstract

We performed ITS sequencing on the culturable endophytic fungi in the fruits of *Cinnamomum migao*, a unique medicinal plant in Southwest China. Our study clarified the relative abundance, diversity, and difference of culturable endophytic fungi in the fruits of *C. migao* from different geographical sources and the correlation between key endophytic fungal metabolites and fruit metabolites.

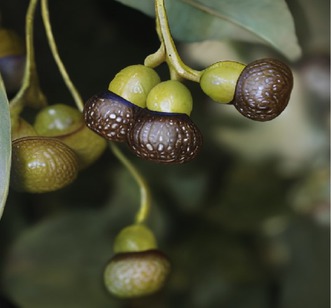

## 1 Introduction

The plant‐associated microbiome plays a pivotal role in plant productivity by enhancing nutrient acquisition, disease suppression, and stress resistance (Trivedi et al. 2020). As the intimate core of plant microbial communities, endophytic microbes inhabit plant tissues—particularly in medicinal plants and—perform vital functions across different developmental stages (Ancheeva et al. 2020). Endophytic fungi exhibit remarkable species diversity and engage in mutualistic relationships with their hosts: plants supply nutrients to endophytes, while the fungi produce secondary metabolites that deter pathogens and stimulate plant growth (Waqar et al. 2024). This growth promotion occurs through direct microbial‐plant interactions, activation of host biosynthetic pathways, or enzymatic conversion of precursors into bioactive compounds (Tao et al. 2024; Wang et al. 2019). Notably, microbial diversity has been shown to enhance plant productivity (Ma et al. 2021; Shen et al. 2022), making the screening of beneficial endophytic fungi a promising strategy for boosting bioactive metabolite synthesis.

While plant microbiomes have been extensively characterized in model species (Chaparro et al. 2014; Kwak et al. 2018; Niu et al. 2017; Turner et al. 2013; Zhang et al. 2019), research has predominantly focused on rhizospheric and phyllospheric communities (Fan et al. 2019; Haney et al. 2015; Liu et al. 2020), with fruit microbiomes remaining largely unexplored (Shade et al. 2017). Emerging studies, however, highlight the functional significance of fruit‐associated microbes in host health (Rybakova et al. 2017; Wassermann et al. 2019). Similarly, seed microbiomes—once overlooked—are now recognized as genotype‐specific microbial reservoirs that vertically transmit core microbiota across generations (Berg and Raaijmakers 2018). These microbes influence critical physiological processes, including seed dormancy, germination, stress adaptation, and disease resistance (Abdelfattah et al. 2023; Berg and Raaijmakers 2018), and contribute to the medicinal quality of host plants (Glynou et al. 2016; Wardle and Lindahl 2014).

*Cinnamomum migao* (Lauraceae), an endemic species to southwestern China, has demonstrated significant therapeutic efficacy in its fruits against gastrointestinal and cardiovascular disorders. Terpenoids and other bioactive compounds underlie its medicinal properties, whose accumulation is influenced by environmental factors and endophytic fungi (Xu et al. 2023). Notably, geographic variations in environmental conditions can lead to divergent microbiota within the same plant species (Zhang et al. 2023).

To address these knowledge gaps, we characterized cultivable endophytic fungi from *C. migao* fruits across geographic regions, analyzing their community structure, diversity, and metabolic profiles. By correlating fungal metabolites with fruit‐derived compounds, we elucidated their roles in bioactive compound accumulation. Furthermore, we identified region‐specific fungi associated with differential metabolite profiles. These findings advance the rational cultivation of *C. migao* for optimized medicinal quality and provide a sustainable approach to harnessing its bioactive compounds.

## 2 Materials and Methods

### 2.1 Sample Collection

**FIGURE 1:**
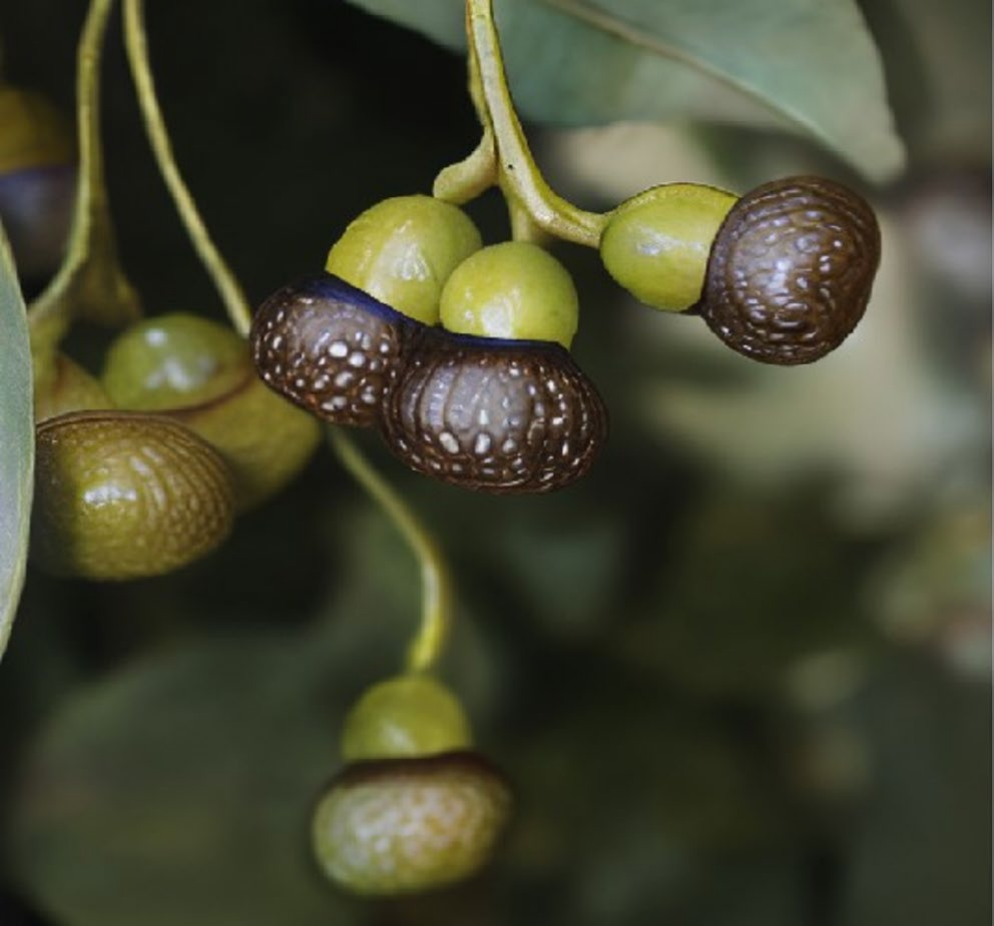
Photograph of *C. migao* fruit samples.

Mature fruit of *C. migao* were collected in October 2021 from 12 populations in three provinces (Guizhou, Yunnan, and Guangxi) in Southern China, which were the main distribution areas of *C. migao* (Table S1). All samples were transported back to the laboratory and stored at 4°C for further processing (Figure 1).

### 2.2 Sample Processing

The surface of the *C. migao* fruit was first rinsed with sterile water and then immersed in 75% ethanol for 1 min. Afterward, the fruit was washed three times with sterile water, immersed in a 5% sodium hypochlorite solution for 3 min, followed by a 30‐s immersion in 75% ethanol, and then washed five times with sterile water. The surface was dried with sterile filter paper, completing the surface disinfection. To ensure that the isolated endophytic fungi were derived from the internal tissues of the *C. migao* fruit, three methods—tissue imprint method (Surface‐sterilized tissue segments from *C. migao* fruits were aseptically transferred onto potato dextrose agar (PDA) plates and allowed to remain in contact with the medium for 3–5 min. Following removal of the tissue segments, the plates were sealed and incubated at 28°C for 7 days. The optimal surface sterilization protocol was identified as the minimal treatment intensity that consistently prevented microbial growth in all three replicate control groups), wash fluid plating method (an aliquot of the final rinse solution was evenly spread onto the surface of a PDA medium and incubated to assess microbial colony formation), and blank plate control method (to verify the sterility of the laminar airflow in the biosafety cabinet, five open PDA plates were exposed during experimental procedures and subsequently incubated under identical conditions) (McInroy and Kloepper 1994; Schulz et al. 1993)—were used to verify the effectiveness of surface disinfection. After disinfection, the *C. migao* fruit was inoculated onto PDA plates and incubated at 28°C in a constant temperature incubator until pure cultures were obtained, resulting in a single fungal strain.

### 2.3 Identification of Endophytic Fungi in *C. Migao* Fruit

The genomic DNA of the endophytic fungi from *C. migao* fruit was extracted using the Fungal Genomic DNA Extraction Kit (centrifuge column type) according to the manufacturer's instructions. The internal transcribed spacer (ITS) region of the fungal ribosomal DNA (Beijing BioTeke Biotechnology Co. Ltd fungal genome) was amplified using the universal fungal primers ITS1‐F (5′‐TCCGTAGGTGAACCTGCGG‐3′) and ITS4‐R (5′‐TCCTCCGCTTATTGATATGC‐3′). The PCR amplification program was as follows: (a) 94°C for 2 min for predenaturation; (b) 34 cycles of 94°C for 30 s (denaturation), 55°C for 30 s (annealing), and 72°C for 30 s (extension); (c) 72°C for 2 min for final extension, followed by storage at −20°C. The PCR products were analyzed by 1% agarose gel electrophoresis, and those that showed a successful amplification pattern were sent for sequencing to Shanghai Qingke Biotechnology Co. Ltd. The sequences were aligned against the GenBank database using the BLAST search tool to identify the endophytic fungi at the molecular level. Morphological identification of the fungi was also performed.

### 2.4 Volatile Oil Composition of *C. Migao* Fruit

The volatile oil composition of *C. migao* fruit was analyzed using gas chromatography–mass spectrometry (GC7890A‐MS5975C; Agilent, USA) and supercritical fluid extraction (Spe‐ed SFE‐2, ASI, USA) (Gere 1983; Song and Ashley 1999; Yang et al. 2010). Powdered *C. migao* fruit (3.00 g), obtained by mechanical grinding (TL‐48R; Shanghai Wanbai Biotechnology Co. Ltd.) and sieving through a 40‐mesh sieve, was accurately weighed and transferred to a 0.5 mL extraction vessel. The pressure was set to 30 MPa, and the temperature was maintained at 300 K for static extraction for 20 min. This was followed by an additional static extraction for 20 min, repeating the process three times. The collected extract was dissolved in 10 mL of ethyl acetate and filtered through a 0.45 μm organic‐phase filter membrane before analysis. The chromatographic conditions included the use of a VF‐5MS (30 m ×0.25 mm, 0.25 μm) capillary column with a temperature program of 50°C held for 1 min, then heated at 3°C·min^−1^ to 120°C, followed by 2°C·min^−1^ to 170°C, and finally 15°C·min^−1^ to 280°C. The sample injection volume was 1 μL, with an injector temperature of 250°C and helium as the carrier gas at a flow rate of 1 mL·min^−1^. The mass spectrometry conditions were set as follows: ion source temperature of 230°C, quadrupole temperature of 150°C, and interface temperature of 280°C. The electron energy was 70 eV, and the mass‐to‐charge ratio (m/z) range was 30–600 amu.

### 2.5 Chemical Composition of Endophytic Fungi From *C. Migao*

A sample of 3–5 g of fungal strain was collected from the upper layer of the culture medium and placed in a solid‐phase microextraction sampling vial. A manual injector with a 2 cm, 50/30 μm DVB/CAR/PDMS Stable Flex fiber was used for headspace extraction at 55°C for 50 min. After extraction, the fiber was immediately inserted into the gas chromatograph injection port (set to 250°C) for thermal desorption. The chromatographic conditions used an HP‐5MS column (60 m × 0.25 mm × 0.25 μm) with an initial temperature of 40°C (held for 2 min), heating at 3.5°C·min^−1^ to 208°C, then at 10°C·min^−1^ to 310°C, for a total runtime of 60.2 min. The injection port temperature was set to 250°C, and helium was used as the carrier gas at a flow rate of 1.0 mL·min^−1^ with a column pressure of 15.98 psi. The mass spectrometer (HP6890/5975C; Agilent, USA) settings were as follows: electron ionization (EI) source, ion source temperature of 230°C, quadrupole temperature of 150°C, electron energy of 70 eV, and a mass‐to‐charge ratio (m/z) range of 29–500 amu.

### 2.6 Diversity Data Statistical Analysis

The diversity, preference, and similarity indices (Ahmed et al. 2008; Zhang et al. 2021) of the endophytic fungi in *C. migao* fruit from different regions were analyzed using indicators such as colonization rate, isolation rate, isolation frequency, diversity index, richness, evenness, and similarity coefficient.

### 2.7 Statistical Analysis

The chemical compositions of volatile oils were identified using mass spectrometry data systems in conjunction with the Nist20 and Wiley275 standard mass spectra. The relative abundance of each compound was determined using the peak area normalization method. SPSS 25 software was employed for one‐way ANOVA, with a significance level of *p* < 0.05 and a VIP value of ≥ 1 to filter differential compounds. Data visualization was performed using Upset and bubble plots on the Lianchuan Biology Cloud Platform (https://www.omicstudio.cn/tool). The Mantel test was performed using the “linkET” package in R, with Pearson's correlation coefficient calculated to assess the association, along with its corresponding statistical significance. Heatmaps were generated using Origin 2021 software.

## 3 Results

### 3.1 Isolation and Identification of Cultivable Endophytic Fungi From *C. Migao* Fruit

A total of 476 strains of endophytic fungi were isolated from the *C. migao* fruit; the fungi were observed and recorded based on their color, colony morphology, odor, pigment production, and growth rate. Some of the fungal colony morphology and their hyphal structure were magnified 40‐fold under the microscope (Figure S1).

### 3.2 Community Composition Characteristics of Cultivable Endophytic Fungi in *C. Migao* Fruit

**FIGURE 2:**
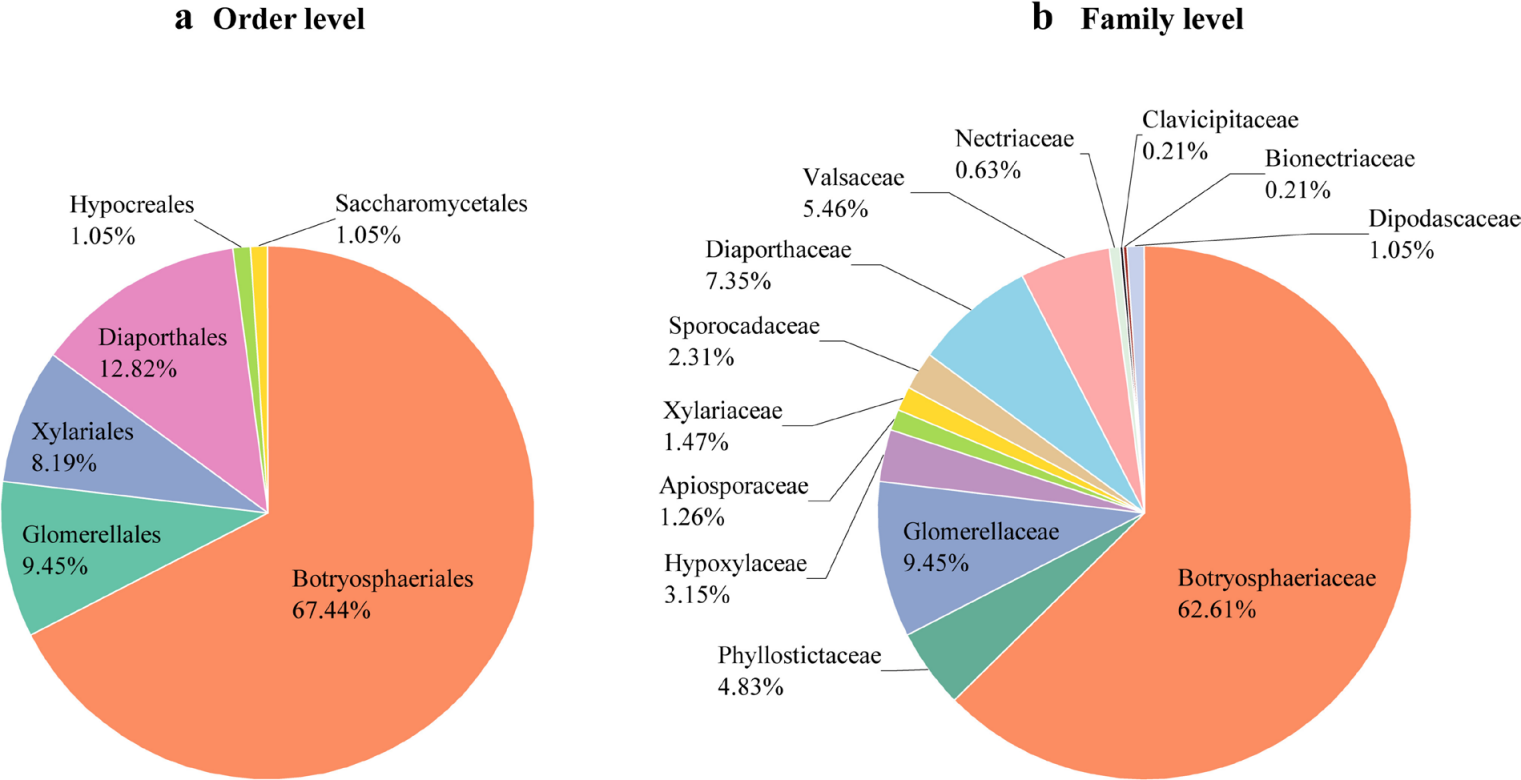
The relative abundance of culturable endophytic fungi in the fruits of *C. migao* at order level (a) and family level (b).

**FIGURE 3:**
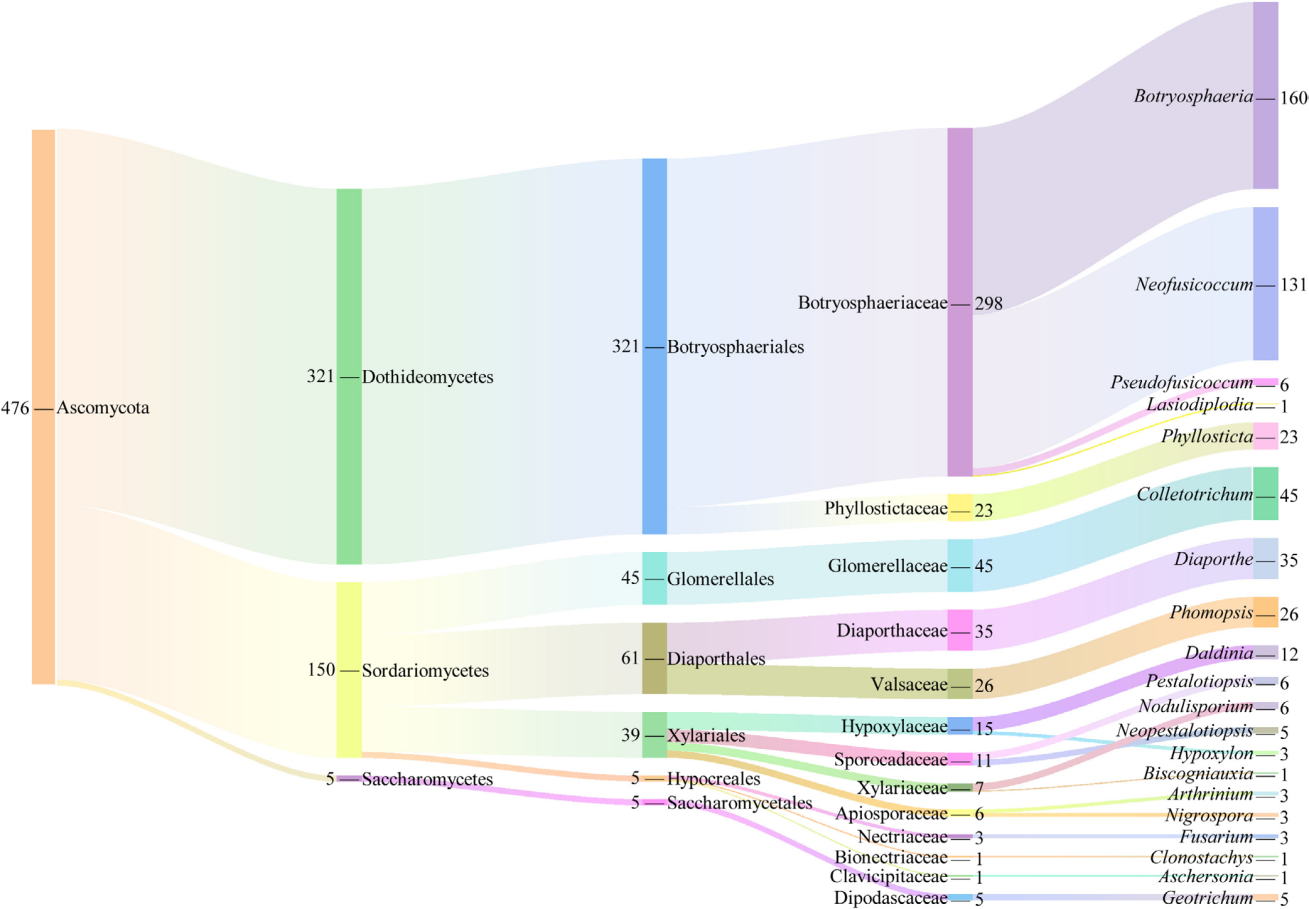
Taxonomic composition of culturable endophytic fungi from different habitats of *C. migao*.

The isolated endophytic fungi were identified based on both morphological and molecular biological characteristics, classified into 1 phylum, 3 classes, 6 orders, 13 families, and 20 genera (Table S2, Figure 2 and Figure 3). At the phylum level, all the endophytic fungi belonged to the Ascomycota. At the class level, they were mainly distributed across three classes: Dothideomycetes, Sordariomycetes, and Saccharomycetes, with Dothideomycetes being the dominant class, accounting for 67.44% of the total strains (321 strains). At the order level, the fungi were predominantly distributed across the orders Botryosphaeriales, Glomerellales, Xylariales, Diaporthales, Hypocreales, and Saccharomycetales, with Botryosphaeriales being the dominant order, comprising 67.44% of the total strains. At the family level, the dominant family was Botryosphaeriaceae, accounting for 62.61% of the total strains. At the genus level, the endophytic fungi in *C. migao* fruit were primarily distributed in the genera *Botryosphaeria* (separation frequency [SF] = 22.22%, isolation rates [IR] = 33.61%) and *Neofusicoccum* (SF = 27.52%, IR = 18.19%), *Colletotrichum* (SF = 9.45%, IR = 6.25%), *Diaporthe* (SF = 7.35%, IR = 4.86%), *Phomopsis* (SF = 5.46%, IR = 3.61%), *Phyllosticta* (SF = 4.83%, IR = 3.19%), *Daldinia* (SF = 2.52%, IR = 1.67%), *Pseudofusicoccum*, *Nodulisporium*, and *Pestalotiopsis* (SF = 1.26%, IR = 0.83%), *Neopestalotiopsis* and *Geotrichum* (SF = 1.05%, IR = 0.69%), *Hypoxylon*, *Arthrinium*, *Nigrospora*, and *Fusarium* (SF = 0.63%, IR = 0.42%), and *Lasiodiplodia*, *Biscogniauxia*, *Aschersonia*, and *Clonostachys* (SF = 0.21%, IR = 0.14%). The dominant genera were *Botryosphaeria* and *Neofusicoccum*. These results indicate that *C. migao* fruit hosts a rich and diverse community of endophytic fungi.

### 3.3 Community Composition of Cultivable Endophytic Fungi From *C. Migao* Fruit in Different Regions and Their Differences

**FIGURE 4:**
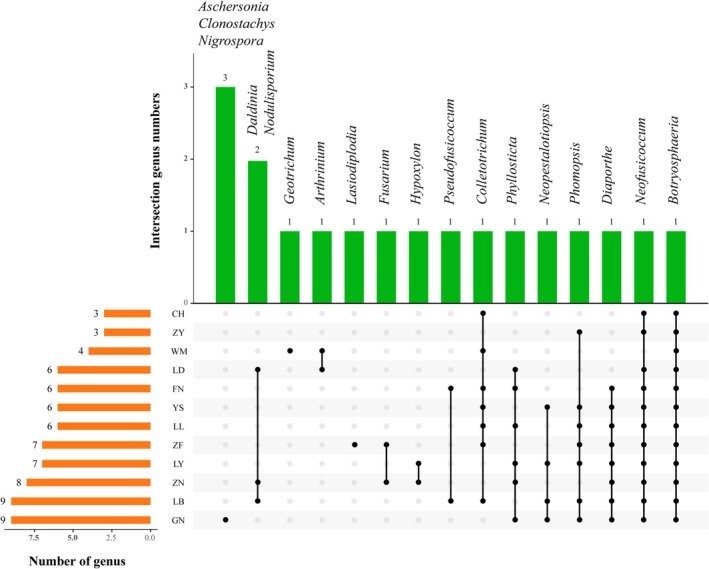
Upset diagram of endophytic fungal communities in the fruits of *C. migao* from different areas at the genus level.

The community composition of endophytic fungi from different regions was analyzed using an Upset plot (Figure 4, Table 1). *Botryosphaeria* was the shared genus across all regions. In addition, some other fungal genera were shared between specific regions. Except for WM, *Neofusicoccum* was a shared genus in the remaining regions. The shared genus in YS, FN, ZF, LY, LB, ZN, and GN was *Diaporthe*; in LD, LL, FN, LY, ZN, and GN, the shared genus was *Phyllosticta*; in ZY, LL, YS, ZF, LY, LB, and GN, the shared genus was *Phomopsis*; in CH, WM, LL, YS, ZF, FN, and LB, the shared genus was *Colletotrichum*; *Daldinia* and *Nodulisporium* were shared in LD, LB, and ZN; *Arthrinium* was shared between WM and LD; *Pseudofusicoccum* was shared in FN and LB, and *Fusarium* was shared between ZF and ZN; *Hypoxylon* was found in both LY and ZN; *Neopestalotiopsis* was shared in LB, YS, LY, and GN. There were also fungi showing certain specificity, found only in specific regions; the GN samples had the most unique genera, which were *Aschersonia*, *Clonostachys*, and *Nigrospora*. WM and ZF each had one unique genus, *Geotrichum* and *Lasiodiplodia*, respectively.

**TABLE 1:** Common and unique genera of endophytic fungi in the fruits of *C. migao* from different origins.

Sampling sites	LB	LD	WM	CH	ZF	ZY	ZN	GN	FN	YS	LL	LY
*Botryosphaeria*	1	1	1	1	1	1	1	1	1	1	1	1
*Colletotrichum*	1	0	1	1	1	0	0	0	1	1	1	0
*Daldinia*	1	1	0	0	0	0	1	0	0	0	0	0
*Diaporthe*	1	0	0	0	1	0	1	1	1	1	1	1
*Neofusicoccum*	1	1	0	1	1	1	1	1	1	1	1	1
*Neopestalotiopsis*	1	0	0	0	0	0	0	1	0	1	0	1
*Nodulisporium*	1	1	0	0	0	0	1	0	0	0	0	0
*Phomopsis*	1	0	0	0	1	1	0	1	0	1	1	1
*Pseudofusicoccum*	1	0	0	0	0	0	0	0	1	0	0	0
*Arthrinium*	0	1	1	0	0	0	0	0	0	0	0	0
*Phyllosticta*	0	1	0	0	0	0	1	1	1	0	1	1
*Geotrichum*	0	0	1	0	0	0	0	0	0	0	0	0
*Fusarium*	0	0	0	0	1	0	1	0	0	0	0	0
*Lasiodiplodia*	0	0	0	0	1	0	0	0	0	0	0	0
*Hypoxylon*	0	0	0	0	0	0	1	0	0	0	0	1
*Aschersonia*	0	0	0	0	0	0	0	1	0	0	0	0
*Clonostachys*	0	0	0	0	0	0	0	1	0	0	0	0
*Nigrospora*	0	0	0	0	0	0	0	1	0	0	0	0

*Note:* 1 indicates shared genera, 0 indicates absence.

### 3.4 Diversity Analysis of Cultivable Endophytic Fungi From *C. Migao* Fruit in Different Regions

The higher the Shannon–Wiener index (H'), the higher the corresponding community diversity and the higher the endophytic fungal community. H' does not have the ability to describe the biodiversity of a community alone, but is suitable for comparing the biodiversity of different communities. The Simpson index (D) is a good index to reflect the dominance of the community; the higher the D, the higher the diversity of endophytic fungi in that community. The diversity index of endophytic fungi in *C. migao* fruits from different production areas is shown in Table 2. Shannon‐Wiener index analysis showed that the diversity of endophytic fungal communities was highest in LB and the diversity of endophytic fungal communities was lowest in ZY. The Simpson index showed that, except for CH, WM, LD, and ZY, there was little difference between samples from other production areas. The dominance of the endophytic fungal community in LB was the greatest, and the dominance of CH was small. Richness index and Evenness index showed that the richness of the endophytic fungal community in GN was the highest, and the distribution of endophytic fungi in LB was the most uniform. Endophytic richness was lowest and most unevenly distributed in ZY. The results showed that the community composition, abundance, and diversity of endophytic fungi in *C. migao* fruits from different production areas were different.

**TABLE 2:** Diversity analysis of culturable endophytic fungi in fruits of *C. migao* from different areas.

Sample sites	Number of genera	Number of species	Shannon–Wiener index (H′)	Simpson index (D)	Richness index	Eveness index
CH	3	8	0.817	0.484	1.911	0.223
LB	9	14	2.064	0.860	3.376	0.536
LD	6	6	1.032	0.528	1.278	0.264
WM	4	6	1.180	0.641	1.517	0.358
ZF	7	14	1.629	0.752	3.415	0.428
ZY	3	4	0.789	0.519	0.819	0.215
ZN	9	11	1.638	0.713	2.769	0.454
LY	7	9	1.516	0.708	2.517	0.477
LL	6	9	1.439	0.708	2.090	0.376
YS	6	8	1.638	0.785	1.939	0.453
GN	10	15	1.760	0.786	3.579	0.450
FN	6	10	1.600	0.770	2.531	0.450

### 3.5 Similarity Analysis of Cultivable Endophytic Fungi From *C. Migao* Fruit in Different Regions

**FIGURE 5:**
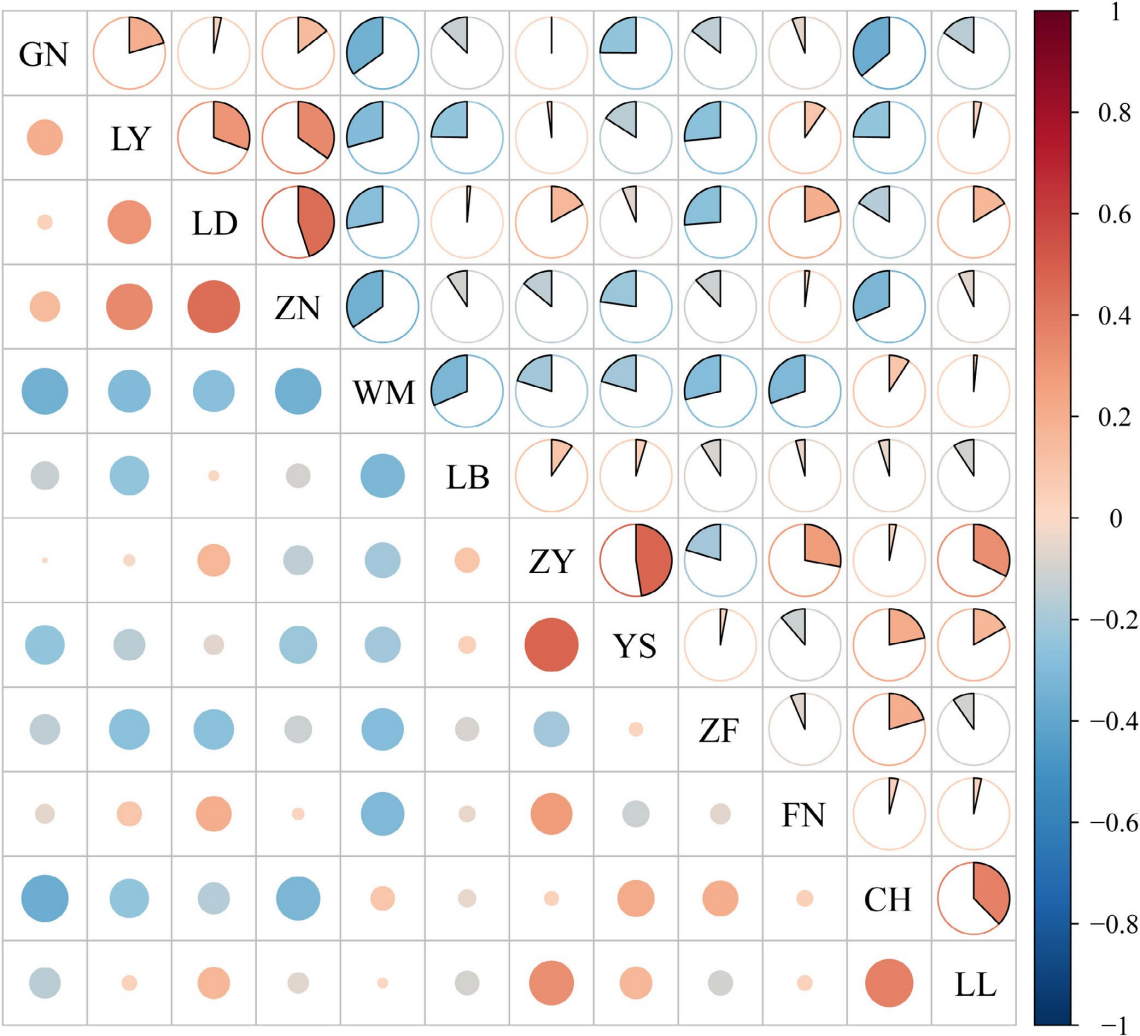
Similarity heat map of culturable endophytic fungi in fruits of *C. migao* from different habitats at genus level.

The similarity coefficient is an important parameter that reflects the degree of similarity in fungal community composition between two different habitats. Based on the principle of the Jaccard similarity coefficient, the results showed that, except for WM and ZF, LD was extremely dissimilar to samples from other production areas; CH was extremely dissimilar to ZN, LY, and GN; LB was extremely dissimilar to ZF, WM, ZN, LY, and GN; except for LL and CH, WM was extremely dissimilar to other samples; except for CH, LL, YS, and FN, ZF was extremely dissimilar to the remaining production areas; ZY was extremely dissimilar to ZF and WM; ZN was extremely dissimilar to YS, CH, LB, WM, and ZF; LY was extremely dissimilar to CH, LB, WM, ZF, and YS; YS was extremely dissimilar to WM, ZN, GN, FN, and LY; GN was extremely dissimilar to FN, CH, LB, WM, and ZF; FN was extremely dissimilar to WM, LL, and YS (Figure 5 and Table S3).

These results indicate that the composition of endophytic fungi in the fruits of *C. migao* varies among different production areas, suggesting significant differences in endophytic fungal composition across sampling sites. The host plant's habitat influences the distribution of endophytic fungi, and the role this variability plays in the formation of medicinal quality warrants further investigation.

### 3.6 Phylogenetic Relationship of Cultivable Endophytic Fungi in *C. Migao* Fruit From Different Origins

**FIGURE 6:**
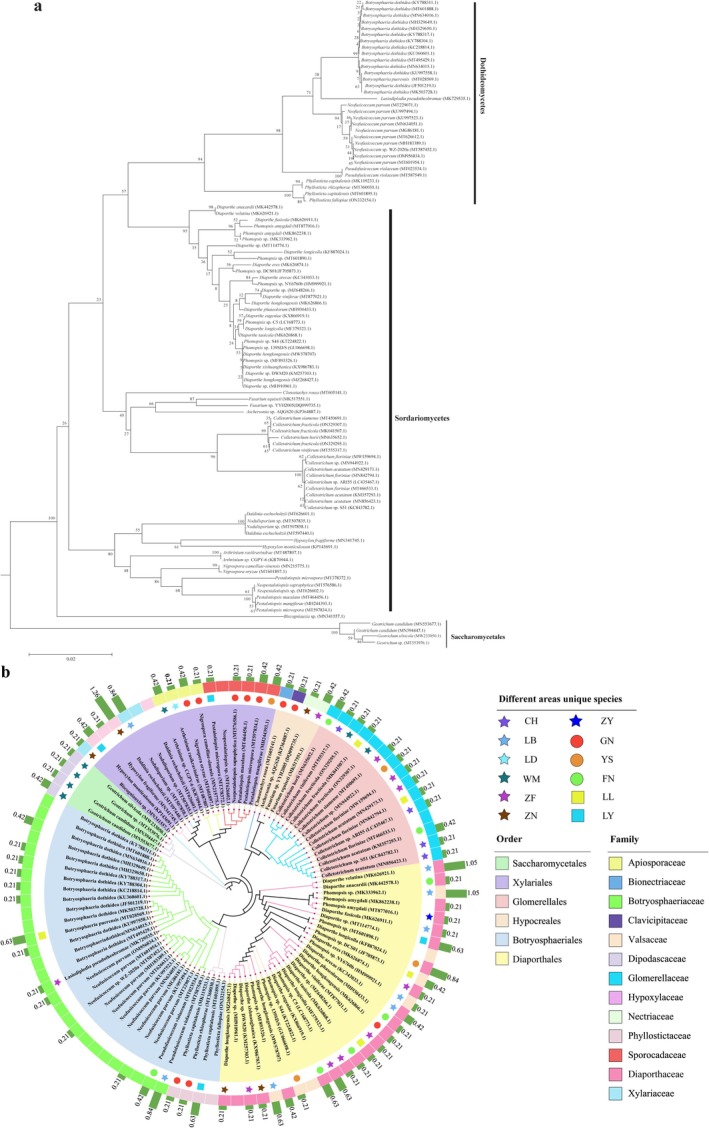
Phylogenetic tree of culturable endophytic fungi in fruits of *C. migao*.The different colors of the inner ring of the developmental tree represent the order of the strain, and the different colors of the outer ring represent the family of the strain; the shape of different colors outside the inner ring indicates the origin of the strain, and the five‐pointed star, round and square all represent that the strain is a unique genus of the origin. The outer circle green histogram shows the relative abundance of the strain in all strains.

**FIGURE 7:**
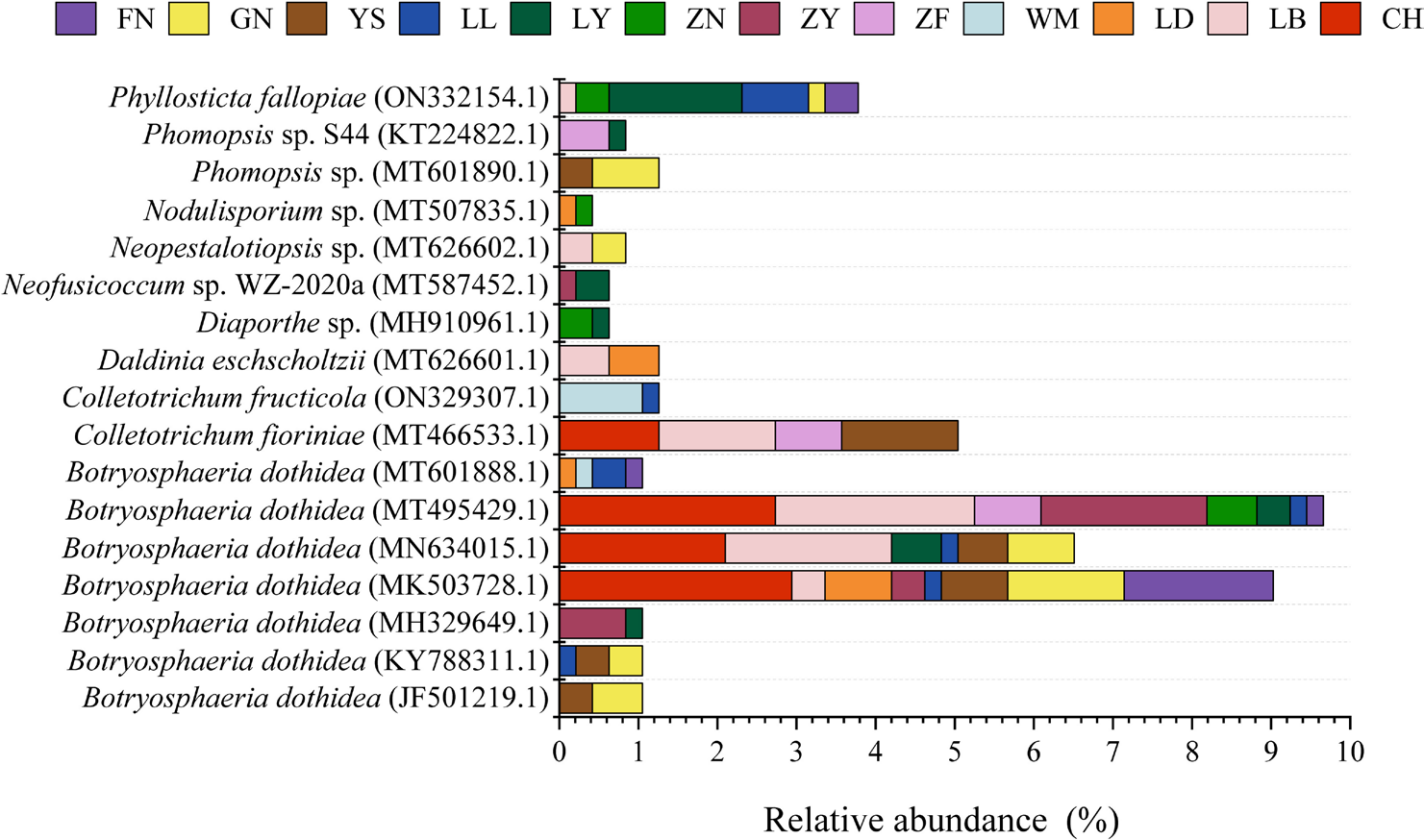
Different areas share species composition and relative abundance.

The phylogenetic analysis of endophytic fungi from *C. migao* fruit was conducted using ITS‐rDNA sequencing. After submitting the sequence results to GenBank and performing a BLAST search, 101 different genotypes were identified. To investigate the phylogenetic relationships of endophytic fungi from various *C. migao* fruit origins, a phylogenetic tree was constructed using the Neighbor‐Joining method in MEGA6.0 (Figure 6). As shown in Figure 6a, *Pseudofusicoccum violaceum* (MT023534.1) was related to *P. violaceum* (MT587549.1), *Daldinia eschscholtzii* (MT626601.1), *Nodulisporium* sp. (MT507835.1), and *Nodulisporium* sp. (MT507858.1), with a bootstrap support of 100%, indicating that these groups have a close phylogenetic relationship. Similarly, *Arthrinium rasikravindrae* (MT487807.1) was highly supported in its relationship with *Arthrinium* sp. CGPY‐6 (KR70944.1). Other fungal groups exhibited bootstrap support ranging from 2% to 99%, reflecting varying degrees of relatedness. Phylogenetically, the endophytic fungi from *C. migao* fruit were primarily distributed within the *Dothideomycetes*, *Botryosphaeriales*, *Botryosphaeriaceae*, and *Botryosphaeria*. *Botryosphaeria dothidea* was found to be a shared species across all regions. Except for the WM region, *Neofusicoccum parvum* was identified as a shared species in all other regions, with the highest abundance observed in the ZY region at 4.20% (Figure 6b and Figure 7).

As shown in Figure 6b, each region had its own unique species. In Guizhou, among all origins, *Colletotrichum* sp. ARI55 (LC435467.1), *Colletotrichum* sp. S51 (KC843782.1), *Colletotrichum acutatum* (MN856423.1), and *Colletotrichum viniferum* (MT555317.1) each account for 0.21% of the total fungal strains. The LB region contained nine unique species: *Phomopsis* sp. (MF893326.1), *Diaporthe arecae* (KC343033.1), *Phomopsis* sp. NY6760b (HM999921.1), *Nodulisporium* sp. (MT507858.1), *Diaporthe velutina* (MK626921.1), *P. violaceum* (MT587549.1), *Diaporthe fusicola* (MK626911.1), *Phomopsis* sp. (MK333962.1), and *Phomopsis amygdali* (MK862238.1). The LD region had only one unique species, *A. rasikravindrae* (MT487807.1), while the WM region was home to six unique species: *Geotrichum silvicola* (MW233050.1), *Geotrichum* sp. (MT353976.1), *Colletotrichum fructicola* (ON329295.1), *Arthrinium* sp. CGPY‐6 (KR70944.1), *Geotrichum candidum* (MN394447.1), and *G. candidum* (MN553677.1). The ZF region had 11 unique species, including *Diaporthe longicolla* (MF379323.1), *C. acutatum* (MN429173.1), *Lasiodiplodia pseudotheobromae* (MK729535.1), *Colletotrichum siamense* (MT450691.1), *Diaporthe viniferae* (MT877021.1), *Diaporthe* sp. DWM20 (KM257303.1), *Diaporthe eres* (MK626874.1), *Colletotrichum fioriniae* (MW159694.1), *Diaporthe hongkongensis* (MW578707), *Phomopsis* sp. C5 (LC168773.1), and *Fusarium equiseti* (MK517551.1). ZN had six unique species, including *Diaporthe xishuangbanica* (KX986783.1), *D. hongkongensis* (MZ268427.1), *Biscogniauxia* sp. (MN341557.1), *Fusarium* sp. YYH2005 (DQ099735.1), *Hypoxylon fragiforme* (MN341745.1), and *D. eschscholtzii* (MT597440.1), while ZY had only one unique species, *P. amygdali* (MT877016.1). In Yunnan, GN had nine unique species: *Clonostachys rosea* (MT605141.1), *D. hongkongensis* (MK626866.1), *Phyllosticta rhizophorae* (MT360030.1), *Phyllosticta capitalensis* (MK119233.1), *Nigrospora camelliae‐sinensis* (MN215775.1), *Neopestalotiopsis saprophytica* (MT576586.1), *Pestalotiopsis maculans* (MT464456.1), *Pestalotiopsis microspora* (MT597834.1), and *Nigrospora oryzae* (MT601897.1). FN had 7 unique species: *C. acutatum* (KM357293.1), *P. violaceum* (MT023534.1), *Colletotrichum horii* (MN635652.1), *Diaporthe phaseolorum* (MH930433.1), *Diaporthe taoicola* (MK626868.1), *Diaporthe anacardii* (MK442578.1), and *Diaporthe eugeniae* (KX866919.1). YS had four unique species, including *D. longicolla* (KF887024.1), *Colletotrichum* sp. (MN944922.1), *Pestalotiopsis mangiferae* (MH244393.1), and *Phomopsis* sp. 139SD/S (GU066698.1). In Guangxi, LL contained four unique species: *Diaporthe* sp. (MZ648266.1), *C. fioriniae* (MN842794.1), *Phomopsis* sp. DCS01 (JF705873.1), and *C. fructicola* (MK041507.1). LY had four unique species: *P. capitalensis* (MT601895.1), *Hypoxylon monticulosum* (KP143691.1), *Diaporthe* sp. (MT114774.1), and *P. microspora* (MT378372.1).

In addition, as shown in Figure 6, there were also shared species across various regions. For example, *C. fructicola* (ON329307.1) was a shared species between WM and LL. *C. fioriniae* (MT466533.1) was a shared species among CH, LB, ZF, and YS, with relatively higher abundance in YS and LB. *D. eschscholtzii* (MT626601.1) was a shared species between LB and LD. *Diaporthe* sp. (MH910961.1) was a shared species between ZN and LY. *Neofusicoccum* sp. WZ‐2020a (MT587452.1) was a shared species between ZY and LY. *Neopestalotiopsis* sp. (MT626602.1) was a shared species between LB and GN. *Nodulisporium* sp. (MT507835.1) was a shared species between LD and ZN. *Phomopsis* sp. (MT601890.1) was a shared species between YS and GN. *Phomopsis* sp. S44 (KT224822.1) was a shared species between ZF and LY. The shared species *Phyllosticta fallopiae* (ON332154.1) was found in LB, ZN, LY, LL, GN, and FN, with LY having the highest relative abundance at 1.68%.

The different colors of the inner ring of the developmental tree represent the order of the strain, and the different colors of the outer ring represent the family of the strain; the shape of different colors outside the inner ring indicates the origin of the strain, and the five‐pointed star, round, and square all represent that the strain is a unique genus of the origin. The outer circle green histogram shows the relative abundance of the strain in all strains.

### 3.7 The Community Structure of Endophytic Fungi in *C. Migao* Fruit and the Correlation Between Endophytic Fungi and Host Chemical Components

#### 3.7.1 Analysis of Chemical Composition Differences in Volatile Oils From *C. Migao* Fruit Across Different Regions

To investigate the chemical composition of volatile oils in *C. migao* fruit from different regions, the chemical components and their relative contents were analyzed using GC–MS for 30 *C. migao* fruit collected from 10 distinct regions. These chemical components were subjected to supervised Orthogonal Partial Least Squares‐Discriminant Analysis (OPLS‐DA) in SIMCA 14.1, generating the corresponding model. The results indicated that the *R* ^*2*^ *x* value, which represented the fitting index for independent variables, was 0.690, while the *R* ^*2*^ *y* value for the dependent variable fitting index was 0.734. The *Q* ^2^(cum) value, which indicated the model's predictive power, was 0.574. Since both *R* ^2^ and *Q* ^2^ exceeded 0.5, the model fit was considered acceptable. The analysis revealed minimal variation between samples from the same region, but significant separation was observed between certain regions, such as FN, LL, LY, and LB, indicating notable differences in their chemical composition. In contrast, regions like GN, LD, WM, ZY, CH, and ZF were grouped more closely together, suggesting smaller differences in chemical composition. The OPLS‐DA model was further validated by 200 permutation tests, with the *Q* ^2^ regression line intersecting the vertical axis at a point below zero, indicating that the model was not overfitted and remained valid for further analysis. This validated model was then used to identify intergroup differential metabolites (Figure S2). From the OPLS‐DA model, 34 peaks with VIP values greater than 1.0 and *P*‐values less than 0.05 were identified as potential biomarkers of chemical composition differences across regions. Ten volatile oil components were selected, each showing significant differences across regions. These components include cis‐sabinol, γ‐eucalyptol, terpinolene, β‐(Z)‐ocimene, bulnesol, bornyl acetate, spathulenol, linalool, thymol, and 2‐Cyclohexen‐1‐one, 4‐hydroxy‐3‐methyl‐6‐(1‐methylethyl)‐, (4R,6S)‐rel‐. These results highlight the substantial differences in volatile oil composition across different regions, providing valuable insights into the chemical markers that could influence the fungal community structure in *C. migao* fruit (Table S2).

#### 3.7.2 Correlation Between Endophytic Fungal Community Structure of *C. Migao* and Host Chemical Composition

**FIGURE 8:**
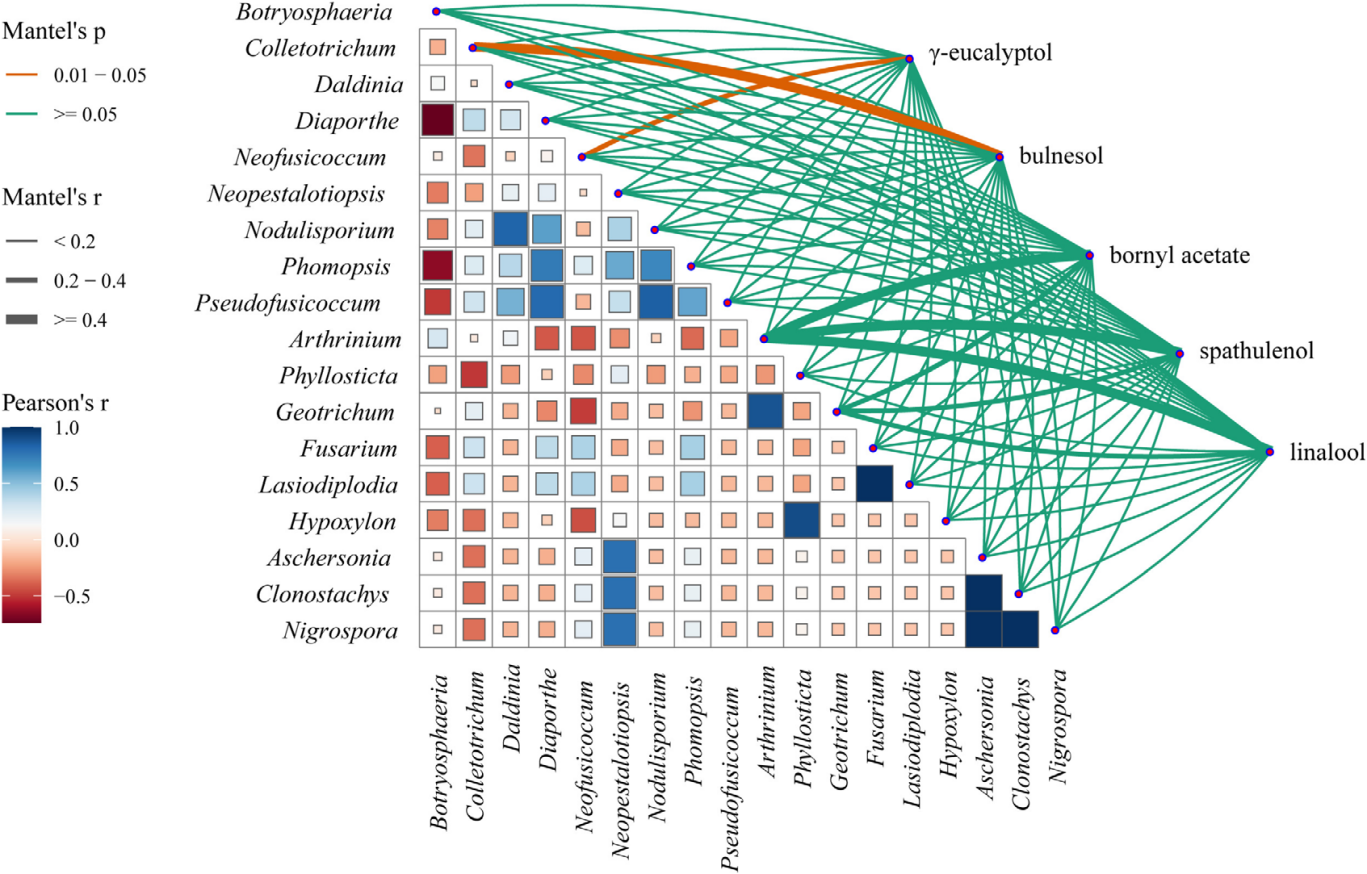
Heatmap of correlation between endophytic fungal diversity and differential metabolites in *C. migao*. A *p*‐value range of 0.01–0.05 denotes statistically significant correlations, with darker color intensities corresponding to stronger Pearson correlation coefficients.

Based on the findings from Section 3.7.1, five differentially abundant compounds showing significant correlations were selected for analysis with endophytic fungi (relative abundance, RF > 1%). As illustrated in Figure 8, all endophytic fungi displayed varying degrees of positive and negative correlations with these compounds. Notably, (+)‐isoguaiacol exhibited significant negative correlations with *Colletotrichum*, *Daldinia*, and *Phyllosticta*, but significant positive correlations with *Clonostachys* and *Nigrospora*. Furthermore, *Phyllosticta* was significantly negatively correlated with eudesmol and strongly positively correlated with linalool. Linalool also showed a highly significant negative correlation with *Neofusicoccum*. The genus *Geotrichum* exhibited mixed correlations, with both positive and negative associations across different fungi. Overall, most endophytic fungal genera correlated negatively with the differential compounds. Exceptions included *Phomopsis*, which was positively correlated solely with bornyl acetate; *Nodulisporium*, which showed a positive correlation only with linalool; and *Daldinia*, which correlated positively only with eudesmol. All other interactions were predominantly negative.

Simple correlation analysis alone cannot fully reflect the actual relationship between independent and dependent variables, so multivariate statistical analysis is needed for further exploration. A linear regression analysis was conducted using the relative frequency of all endophytic fungi as the independent variable and the peak area of differential compounds as the dependent variable. The regression equation was subjected to significance testing, and all tests were significant, indicating that the equation was effective (Table S4). The results showed that the content of γ‐eucalyptol was mainly influenced by *Botryosphaeria* and *Pseudofusicoccum*, both of which exhibited positive correlations. The content of eucalyptol alcohol was mainly influenced by *Daldinia*, *Nodulisporium*, *Botryosphaeria*, and *Phyllosticta*, and eucalyptol alcohol showed negative correlations with *Nodulisporium* and *Phyllosticta*, and positive correlations with *Daldinia* and *Botryosphaeria*. The impact of these fungi on eucalyptol alcohol content followed the order: *Daldinia*, *Botryosphaeria*, *Phyllosticta*, *Nodulisporium*. Bornyl acetate content was primarily influenced by *Daldinia*, *Pseudofusicoccum*, and *Botryosphaeria*, consistent with the results from simple correlation analysis. Linalool content was mainly influenced by *Botryosphaeria* and *Neofusicoccum*, both of which showed negative correlations. In conclusion, the main endophytic fungal communities influencing the content of the five differential compounds in this study were *Daldinia*, *Nodulisporium*, *Pseudofusicoccum*, and *Botryosphaeria*. The endophytic fungi influencing the content of the five differential compounds varied between simple correlation analysis and multivariate statistical analysis.

#### 3.7.3 Comparison of Endophytic Fungal Metabolites and Fruit Volatile Oil Chemical Composition

**FIGURE 9:**
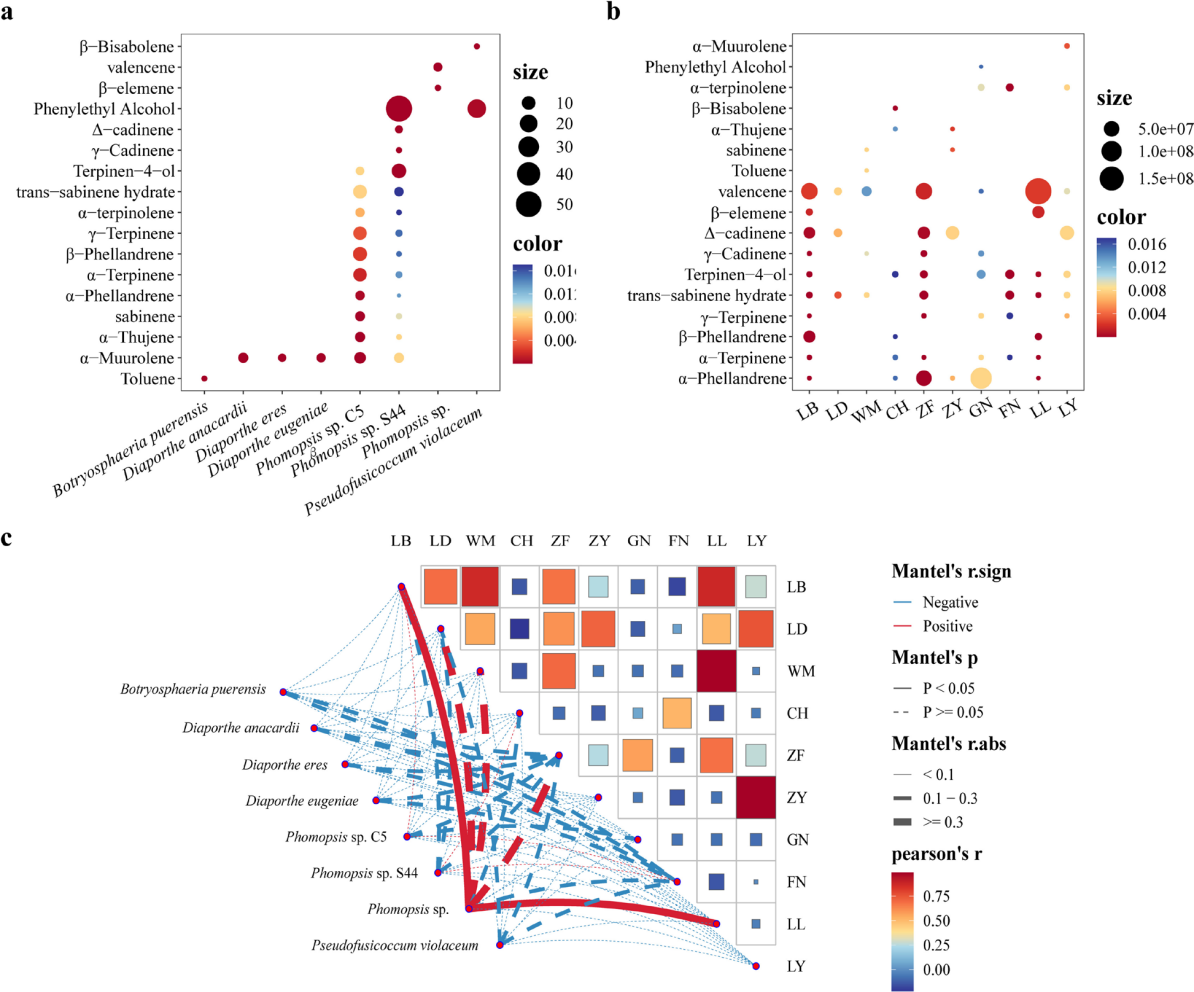
Heatmap of correlation of shared components between *C. migao* fruit and *C. migao* culturable endophytic fungi. (a) Bubble plot of relative metabolite content of culturable endophytic fungi in *C. migao*; (b) Bubble plot of volatile compound content in *C. migao* fruits; (c) Correlation heatmap.

Metabolite profiling of endophytic fungi revealed that eight strains produced chemical constituents identical to those found in the volatile oils of *C. migao* fruit (Figure 9). Among these, *Botryosphaeria puerensis* (MT028569.1) contained toluene, though at a low relative abundance (0.583%). The strains *Diaporthe anacardia* (MK442578.1), *D. eres* (MK626876.1), and *D. eugeniae* (KX866919.1) all produced α‐muurolene, with relative concentrations of 4.272%, 2.150%, and 3.088%, respectively. *Phomopsis* sp. (MK333962.1) synthesized β‐elemene (0.760%) and valencene (2.909%), while *P. violaceum* (MT587549.1) produced β‐bisabolene (0.611%) and phenylethyl alcohol (22.700%), the latter being its most abundant metabolite. Additionally, *Phomopsis* sp. C5 (LC168773.1) and *Phomopsis* sp. S44 (KT224822.1) shared multiple volatile compounds with *C. migao* fruit, including α‐muurolene, α‐thujene, sabinene, α‐phellandrene, α‐terpinene, β‐phellandrene, γ‐terpinene, α‐terpinolene, *trans*‐sabinene hydrate, and terpinen‐4‐ol. Notably, *Phomopsis* sp. S44 also contained γ‐cadinene, δ‐cadinene, and phenylethyl alcohol, with phenylethyl alcohol reaching a remarkably high relative concentration of 52.494%, making it the dominant compound in this strain.

Correlation analysis between the metabolites of culturable endophytic fungi and *C. migao* fruit volatiles indicated a significant positive association between *Phomopsis* sp. and the LB and LL production regions. Furthermore, valencene and β‐elemene identified in *Phomopsis* sp. were present at elevated levels in both LB and LL, suggesting a potential biosynthetic link between these fungi and the host plant's volatile profile.

## 4 Discussion

Consistent with the report by Chen et al. (2021) and Wang et al. (2019), the endophytic fungal communities in the fruits, rhizosphere, and root endosphere of *C. migao* were predominantly composed of Ascomycota. It is believed that *Ascomycete* fungi, due to their faster evolutionary speed and adaptability, result in a higher diversity of fungal communities (Challacombe et al. 2019; Wang et al. 2010). Ascomycetes are also considered a key resource influencing the content of bioactive compounds in *Sophora flavescens* (Ju et al. 2024). Studies have shown that the composition of plant endophytic fungal communities is influenced by various factors such as plant tissue, geographic location, growth conditions, temperature, climate, and rainfall. These unique geographic environments and growth conditions shape the distinctive fungal communities and structural characteristics within plants (Joshee et al. 2009; Mishra et al. 2012). Due to differences in climate, soil, and other factors across regions, the fungal community structure in medicinal plants varies, which in turn affects the quality of the medicinal materials (Chen et al. 2021; Dang et al. 2021; Yang et al. 2024).

Endophytic fungi play a crucial role in maintaining the internal stability of plants and supporting their growth and development. Fungal genera such as *Nigrospora* and *Clonostachys* may appear less frequently in the endophytic fungal community of *C. migao* fruits compared to other genera, but they are of significant importance. For example, *Nigrospora*, in addition to being a major pathogen causing leaf spot diseases in plants, produces compounds like polyketides, steroids, and alkaloids, which possess antimicrobial, antioxidant, antiviral, and anticancer activities (Ukwatta et al. 2019). *C. rosea*, a potential biocontrol agent, plays an important role in controlling fruit rot diseases and promoting plant growth (Iqbal et al. 2018).

It has been reported that the coevolution of endophytic fungi and their host plants leads to the production of secondary metabolites, which play a crucial role in the adaptation between the fungi and their host plants as well as their response to different environments (Ren and Dai 2013). The levels of certain compounds in plants can be influenced by multiple endophytic fungi (Pandey et al. 2018; Velasco et al. 2021). Endophytic fungi can enhance the production of secondary metabolites in host plants. For example, a study found that an endophytic fungus, *Umbelopsis dimorpha*, isolated from the medicinal plant *Kadsura angustifolia*, not only produced compounds similar to those of its host but also promoted the production of triterpenoids or sesquiterpenoids in the plant (Qin et al. 2018).

In this study, it was found that the metabolic products of *Phomopsis* sp. C5 and *Phomopsis* sp. S44 contained a higher number of compounds identical to those found in the volatile oils of *C. migao* fruits; notably, α‐terpinolene, which is one of the differential metabolites of *C. migao* fruit, exhibits excellent antioxidant activity (Kim et al. 2004). Additionally, previous research had shown that the secondary metabolites from *Phomopsis* sp. fermentation had good antimicrobial effects against various plant pathogens; furthermore, the high relative contents of β‐phellandrene, α‐terpinene, and sabinene in these two strains may be related to their activity against *Candida albicans* (Liu et al. 2025; Rukachaisirikul et al. 2008; Yang et al. 2021).

Our study also found that valencene and β‐elemene, two chemical components identified from *Phomopsis* sp. (Fields and Friman 2022), not only showed a significant correlation with two geographical locations (LB and LL) but also had higher contents; this indicates a relationship between geographical environment and endophytic fungi. Finally, although the relative content of β‐bisabolene and β‐elemene in *P. violaceum* and *Phomopsis* sp. is less than 1%, these compounds are of considerable significance; studies have found that β‐bisabolene exhibits specific cytotoxicity against human and mouse mammary tumor cells, while β‐elemene has antiproliferative effects on cancer cells and can induce apoptosis in tumor cells (Chen et al. 2020; Yeo et al. 2016).

The chemical composition of plants is influenced by more than one endophytic fungus. Some chemical constituents are affected by multiple endophytic fungi, showing that plant secondary metabolites may not be solely influenced by a single fungal species; instead, one or more fungal communities likely work together to impact the synthesis or yield of the host's metabolites. Similarly, the composition and distribution of endophytic fungi within the host may be influenced by the synergistic effects of various chemical compounds.

In addition, Wang et al. (2019) found that linalool, α‐terpineol, and β‐caryophyllene isolated from four spice plants of the Lauraceae family exhibited both toxicity and repellency against *Tribolium castaneum* and *Liposcelis bostrychophila* . These fungal strains exhibit distinct biological effects on plant hosts and represent cultivable endophytes of *C. migao* with potential for biotechnological applications.

A deeper understanding of the interaction between the endophytic fungal community structure and the chemical constituents of host plants could lead to improvements in the quality of medicinal plants; this insight could also help identify new endophytic fungal resources to enhance the quality of medicinal materials. The complexity of these interactions highlights the importance of studying both the fungal communities and the secondary metabolites.

## 5 Conclusion

Our study elucidates the relative abundance, diversity, and differences of culturable endophytic fungi in *C. migao* fruits from different geographical sources. Our study also compares some key endophytic fungal metabolites with the fruit's own metabolites, enhancing our understanding of the role of culturable endophytic fungi in *C. migao* fruit growth. These findings highlight the varying degrees of contribution by endophytic fungi during the growth of *C. migao*, offering significant insights into improving the quality of *C. migao* medicinal materials and guiding the selection of high‐quality medicinal resources.

## Author Contributions

**Shuang‐Yan Hu:** formal analysis (equal), investigation (equal), writing – original draft (equal). **Lan Zhang:** investigation (equal), writing – review and editing (equal). **Xin Yang:** data curation (equal), formal analysis (equal), writing – review and editing (equal). **Qing‐Wen Sun:** conceptualization (equal), funding acquisition (equal), supervision (equal), writing – review and editing (equal). **Jing‐Zhong Chen:** conceptualization (equal), funding acquisition (equal), project administration (equal), supervision (equal), writing – original draft (equal), writing – review and editing (equal).

## Conflicts of Interest

The authors declare no conflicts of interest.

## Supporting information


**Figure S1.** Morphology of some fungi.
**Figure S2.** Orthogonal partial least squares discriminant analysis score plot (a) and permutation test (b) of the common chemical components of volatile oil from *C. migao* fruits of different producing areas.
**Table S1.** Sampling site of the fruit of *C. migao*.
**Table S2.** Community composition of culturable endophytic fungi from *C. migao* fruit.
**Table S3.** Comparison of similarity coefficient of culturable endophytic fungi in fruits of *C. migao* from different areas.
**Table S4.** The composition and regression equation of endophytic fungi affecting the content of 10 different compounds were established.

## Data Availability

All the amplicon sequences were deposited in the GenBank database of the National Center for Biotechnology Information (NCBI), and the biological project number was SUB14904716. Data are available in Supporting Information.
