# Supplementary material for: The Accumulation of Chemical Components of Volatile Oil in Cinnamomum migao Fruit Is Related to Endophytic Fungi
Source: Ecol Evol. 2025 Jun 9;15(6):e71548. doi: 10.1002/ece3.71548 (PMC12146584; doi:10.1002/ece3.71548)
Supplement: Supplementary file 1 — Figure S1. Morphology of some fungi. Figure S2. Orthogonal partial least squares discriminant analysis score plot (a) and permutation test (b) of the common chemical components of volatile oil from C. migao fruits of different producing areas. Table S1. Sampling site of the fruit of C. migao. Table S2. Community composition of culturable endophytic fungi from C. migao fruit. Table S3. Comparison of similarity coefficient of culturable endophytic fungi in fruits of C. migao from different areas. Table S4. The composition and regression equation of endophytic fungi affecting the content of 10 different compounds were established. [file ECE3-15-e71548-s001.docx]

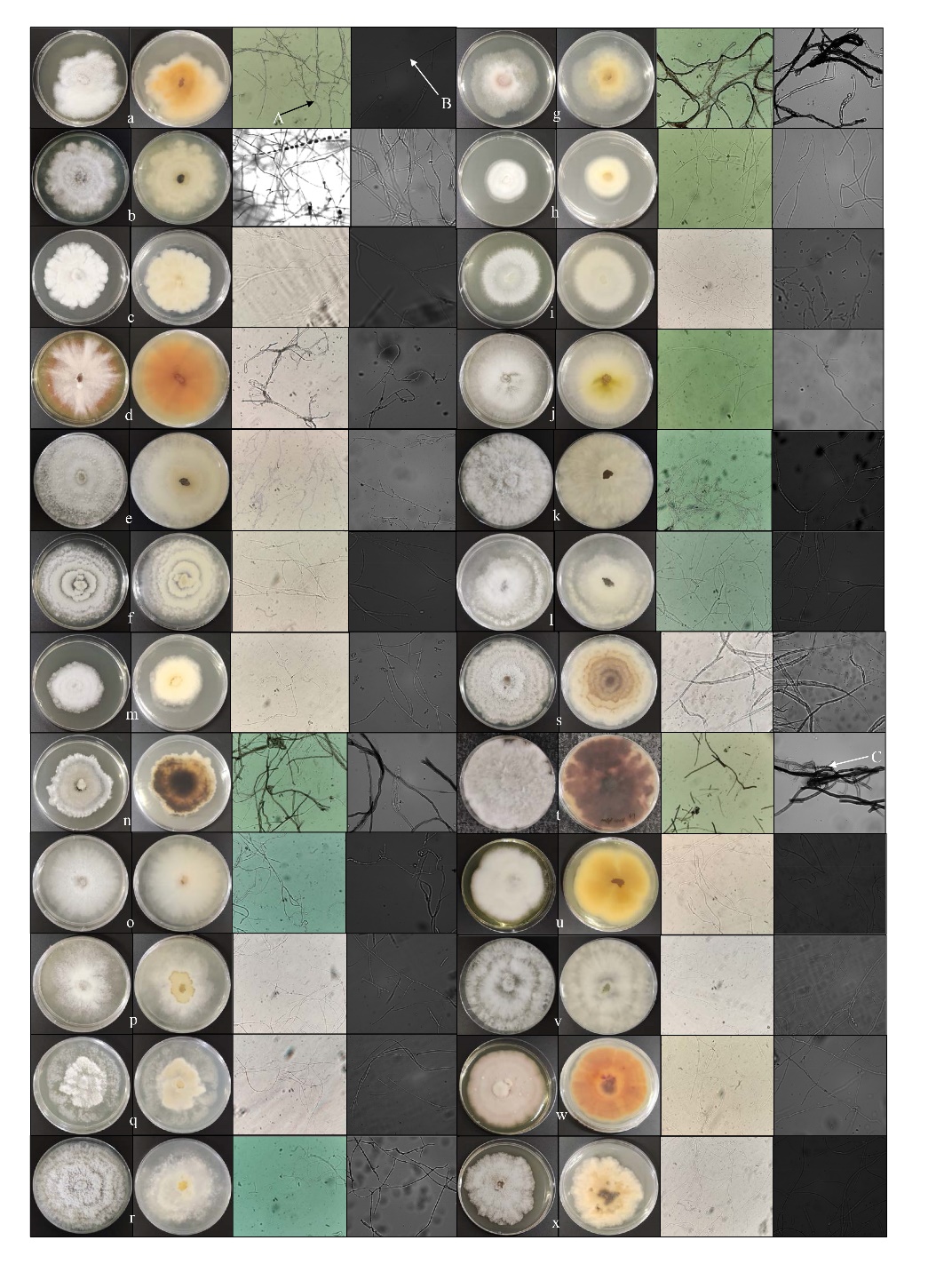


Figure S1 Morphology of some fungi

a ~ x: colonies, A and B: mycelium morphology at 40-fold magnification; C: cords


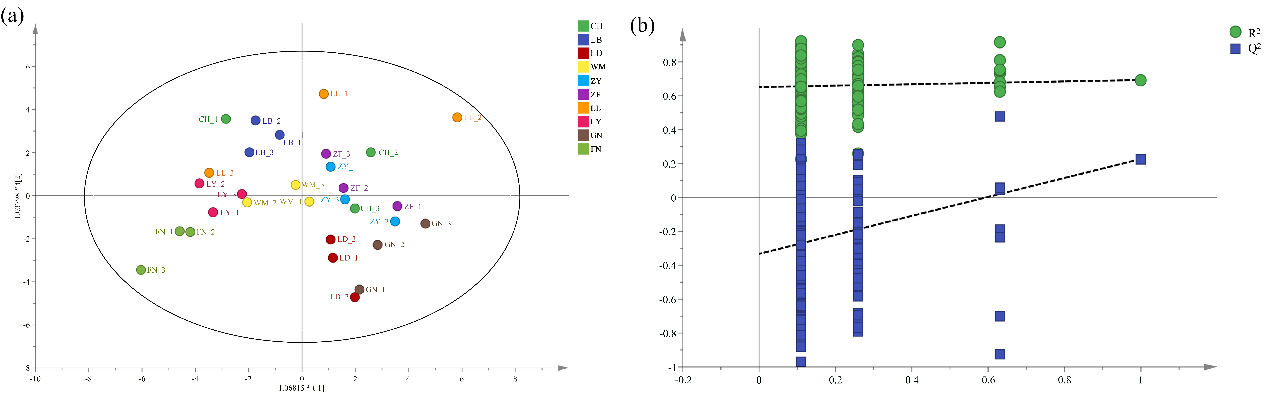


Figure S2 Orthogonal partial least squares discriminant analysis score plot (a) and permutation test (b) of the common chemical components of volatile oil from *C. migao* fruits of different producing areas

**Table S1 Sampling site of the fruit of *C. migao***

| Sampling sites | | Longitude/E | Latitude/N | Altitude/m |
| --- | --- | --- | --- | --- |
| Guizhou | Libo County（LB） | E 106°52'59" | N 25°24'51" | 739 |
|  | Luodian County（LD） | E 106°35'40" | N 25°17'20" | 805 |
|  | Wangmo County（WM） | E 106°16'33" | N 25°4'7" | 985 |
|  | Ceheng County（CH） | E 106°6'17" | N 24°50'56" | 873 |
|  | Zhenfeng County（ZF） | E 105°40'15" | N 25°19'58" | 1019 |
|  | Ziyun Miao and Buyi Autonomous County（ZY） | E 106°18'32" | N 25°22'40" | 968 |
|  | Zhenning Buyei and Miao Autonomous County（ZN） | E 105°50'1" | N 25°50'22" | 962 |
| Yunnan | Guangnan County（GN） | E 104°52'4" | N 24°19'34" | 910 |
|  | Funing County（FN） | E 105°30'56" | N 23°28'19" | 1226 |
|  | Yanshan County（YS） | E 104°19'59" | N 23°36'26" | 1546 |
| Guangxi | Multinational Autonomous County of Longlin（LL） | E 105°29'27" | N 24°37'41" | 1325 |
|  | Leye County（LY） | E 106°37'49" | N24°57'15" | 886 |

**Table S2 Community composition of culturable endophytic fungi from *C. migao* fruit**

| Phylum | Class | Order | Family | Genus | Number | Isolation Rates （IR）（%） | Separation Frequency  SF（%） |
| --- | --- | --- | --- | --- | --- | --- | --- |
| Ascomycota | Dothideomycetes | Botryosphaeriales | Botryosphaeriaceae | *Botryosphaeria* | 160 | 22.22 | 33.61 |
|  |  |  |  | *Lasiodiplodia* | 1 | 0.14 | 0.21 |
|  |  |  |  | *Neofusicoccum* | 131 | 18.19 | 27.52 |
|  |  |  |  | *Pseudofusicoccum* | 6 | 0.83 | 1.26 |
|  |  |  | Phyllostictaceae | *Phyllosticta* | 23 | 3.19 | 4.83 |
|  | Sordariomycetes | Glomerellales | Glomerellaceae | *Colletotrichum* | 45 | 6.25 | 9.45 |
|  |  | Xylariales | Hypoxylaceae | *Daldinia* | 12 | 1.67 | 2.52 |
|  |  |  |  | *Hypoxylon* | 3 | 0.42 | 0.63 |
|  |  |  | Apiosporaceae | *Arthrinium* | 3 | 0.42 | 0.63 |
|  |  |  |  | *Nigrospora* | 3 | 0.42 | 0.63 |
|  |  |  | Xylariaceae | *Biscogniauxia* | 1 | 0.14 | 0.21 |
|  |  |  |  | *Nodulisporium* | 6 | 0.83 | 1.26 |
|  |  |  | Sporocadaceae | *Pestalotiopsis* | 6 | 0.83 | 1.26 |
|  |  |  |  | *Neopestalotiopsis* | 5 | 0.69 | 1.05 |
|  |  | Diaporthales | Diaporthaceae | *Diaporthe* | 35 | 4.86 | 7.35 |
|  |  |  | Valsaceae | *Phomopsis* | 26 | 3.61 | 5.46 |
|  |  | Hypocreales | Nectriaceae | *Fusarium* | 3 | 0.42 | 0.63 |
|  |  |  | Clavicipitaceae | *Aschersonia* | 1 | 0.14 | 0.21 |
|  |  |  | Bionectriaceae | *Clonostachys* | 1 | 0.14 | 0.21 |
|  | Saccharomycetes | Saccharomycetales | Dipodascaceae | *Geotrichum* | 5 | 0.69 | 1.05 |

**Table S3 Comparison of similarity coefficient of** **culturable endophytic fungi in fruits of *C. migao* from different areas**

| Sample sites | CH | LB | LD | WM | ZF | ZY | ZN | LY | LL | YS | GN | FN |
| --- | --- | --- | --- | --- | --- | --- | --- | --- | --- | --- | --- | --- |
| CH | 1 | 0.273 | 0.286 | 0.286 | 0.363 | 0.333 | 0.211 | 0.235 | 0.471 | 0.375 | 0.174 | 0.333 |
| LB |  | 1 | 0.300 | 0.100 | 0.214 | 0.333 | 0.240 | 0.174 | 0.261 | 0.273 | 0.207 | 0.250 |
| LD |  |  | 1 | 0.167 | 0.200 | 0.400 | 0.471 | 0.400 | 0.400 | 0.286 | 0.286 | 0.375 |
| WM |  |  |  | 1 | 0.100 | 0.200 | 0.118 | 0.133 | 0.267 | 0.143 | 0.095 | 0.125 |
| ZF |  |  |  |  | 1 | 0.222 | 0.240 | 0.174 | 0.260 | 0.273 | 0.207 | 0.250 |
| ZY |  |  |  |  |  | 1 | 0.267 | 0.308 | 0.462 | 0.500 | 0.316 | 0.429 |
| ZN |  |  |  |  |  |  | 1 | 0.400 | 0.300 | 0.211 | 0.308 | 0.286 |
| LY |  |  |  |  |  |  |  | 1 | 0.333 | 0.235 | 0.333 | 0.316 |
| LL |  |  |  |  |  |  |  |  | 1 | 0.353 | 0.250 | 0.316 |
| YS |  |  |  |  |  |  |  |  |  | 1 | 0.174 | 0.222 |
| GN |  |  |  |  |  |  |  |  |  |  | 1 | 0.240 |
| FN |  |  |  |  |  |  |  |  |  |  |  | 1 |

**Table S4 The composition and regression equation of endophytic fungi affecting the content of 10 different compounds were established**

| Compounds | Regression equation | F value of the equation |
| --- | --- | --- |
| γ-eucalyptol (Y_1_) | Y_1_=20229606.127+273714480.690544X_3_+19558161.885X_4_ | 2.790* |
| bulnesol (Y_2_) | Y_2_=49950459.612+70507066.196X_3_ | 2.909* |
| spathulenol (Y_3_) | Y_3_=20077732.466+15588638.115X_1_-22949441.442X_2_+1074305.754X_4_-5268912.392X_5_ | 2.975* |
| bornyl acetate (Y_4_) | Y_4_=15213506.184+18916243.503X_1_-15621651.623X_3_-2950594.049X_4_ | 3.417** |
| linalool (Y_5_) | Y_5_=116763824.281-8573377.359X_4_-17166923.172X_7_ | 2.864* |

X_1_: *Daldinia*; X_2_: *Nodulisporium*; X_3_: *Pseudofusicoccum*; X_4_: *Botryosphaeria*; X_5_: *Phyllosticta*; X_6_: *Geotrichum*; X_7_: *Neofusicoccum*
